# Neck Dissection in cT3/T4 Mucoepidermoid Carcinoma of the Oral Cavity and Oropharynx

**DOI:** 10.1007/s12070-024-04808-3

**Published:** 2024-06-27

**Authors:** Rushi Patel, Aman M. Patel, Lucy Revercomb, Amy Patel, Christopher C. Tseng, Richard Chan Woo Park

**Affiliations:** 1 Department of Otolaryngology – Head and Neck Surgery, Cleveland Clinic College of Medicine, Cleveland, OH USA; 2 Department of Otolaryngology - Head and Neck Surgery, Rutgers New Jersey Medical School, 185 S Orange Ave, Newark, NJ 07103 USA; 3 Lake Erie College of Osteopathic Medicine, Elmira, NY USA; 4 Department of Otolaryngology - Head and Neck Surgery, Pennsylvania State University College of Medicine, Hershey, PA USA

**Keywords:** Mucoepidermoid Carcinoma, Oral Cavity, Oropharynx, NCDB, Survival, Neck Dissection

## Abstract

Previous research has reported high occult nodal metastases rates for T3/T4 mucoepidermoid carcinoma (MEC) of the oropharynx (OP) and oral cavity (OC). Our study evaluates if there is a benefit of neck dissection (ND) in these patients. The 2004–2016 National Cancer Database was queried for cases of adult MEC of the OC and OP. Patients with clinical T3/T4 disease were included while those with metastatic disease were excluded. Patients were divided into two cohorts: those treated with and without ND. Univariate chi-square, Kaplan-Meier, and multivariable Cox regression analyses were implemented. A total of 243 patients met inclusion criteria, of which 79 (32.5%) underwent ND. The majority of patients were less than 60 years old (60.1%), White (76.2%), and male (53.5%). 92 (37.9%) patients had clinically node-positive (cN+) disease. ND patients had higher rates of cN + disease (53.2% vs. 30.5%, *p* = 0.002). Of patients undergoing ND, 35 (44.3%) had cN0 disease while 42 (53.2%) had cN + disease. ND patients more commonly had grade III/IV tumors (45.1% vs. 23.4%, *p* = 0.002). Upon examination of dissected nodes, 20.3% of cN0 patients undergoing ND were found to have occult nodal metastases. There was no significant difference in 5-year overall survival between patients with and without ND (61.8% vs. 53.6%, *p* = 0.610), even on multivariable Cox analysis (hazard ratio: 1.52, 95% confidence interval: 0.73–3.18, *p* = 0.269). Our study found patients with cN0 MEC of the OC and OP have a high rate (20.3%) of occult nodal metastasis. In this cohort, patients with ND were not found to have improved survival, possibly due to statistical underpowering. Further research is needed to evaluate the indications and benefit of ND for this rare tumor presentation.

## Keypoints

Previous research has reported high occult nodal metastases rates for T3/T4 mucoepidermoid carcinoma (MEC) of the oropharynx (OP) and oral cavity (OC).

Neck dissection patients had higher rates of cN + disease and more commonly had grade III/IV tumors.

Patients with MEC of the OP and OC have a high rate of occult nodal metastasis, suggesting neck dissection should be given strong consideration for these patients.

There was no significant difference in 5-year overall survival between patients treated with and without neck dissection.

## Introduction

Salivary gland cancers (SGCs) represent 3–6% of all head and neck malignancies [1, 2]. Approximately 20% of SGCs arise in the minor salivary glands which are located in the upper aerodigestive tract and sinonasal cavity, comprising sites such as the palate and oral mucosa [2–4]. Tumors arising from these minor glands have a higher likelihood of malignancy than tumors of the major salivary glands [2, 4, 5]. While several histological subtypes of SGCs exist, mucoepidermoid carcinoma (MEC) is the most common minor salivary gland cancer (MiSGC), accounting for 16.5-45% of reported cases [4, 6].

Neck dissection is a potential treatment option performed to prophylactically remove lymph nodes that may be harboring cervical lymph node metastases [7–9]. Occult nodal metastasis in SGC occurs in 8–50% of patients, with high-grade histological subtypes having the highest incidence [10–12]. In the first report of occult nodal metastasis rates of MEC of the oral cavity (OC) and oropharynx (OP), Ellis et al. similarly found higher rates of occult disease in cT3/T4 tumors, suggesting potential benefit of prophylactic neck dissection in these patients [13]. Recent studies evaluating the benefit of elective neck dissection in SGC have recommended elective neck treatment for patients with high-grade histology, cT3/T4 tumors, or carcinoma arising from the submandibular, sublingual, or minor salivary glands [10–12, 14–16]. However, these studies often encompass a variety of histological subtypes and primary sites, limiting disease-specific information [10, 11]. The purpose of this study is to evaluate the benefit of neck dissection on survival in cT3/T4 MEC of the OC and OP. Given the low incidence of these tumors, our study leverages the National Cancer Database (NCDB) to ascertain this question.

## Methods

The 2004–2016 NCDB was queried for all cases of adult MEC of the OC and OP. *International Classification of Diseases for Oncology*, 3rd edition (ICD-O-3) codes were used to identify cases. MEC was identified with the following histology code: 8430. Primary sites of the OC and OP that were included were: C01.9, C02.2, C02.3, C02.4, C03.0, C03.1, C03.9, C04.0, C04.1, C04.8, C04.9, C05.0, C05,1, C05.2, C06.0, C06.1, C06.2, C09.0, C09.1, C09.8, C09.9, C10.2, C10.3, and C10.9. Only patients with cT3/T4 disease were included in analysis. Patients with multiple cancer diagnoses, metastatic disease, or unknown scope of regional lymph node surgery were excluded. As data from the NCDB is fully de-identified, our study was considered exempt from review by the Rutgers New Jersey Medical School Institutional Review Board.

Variables included in the analysis were age, race, sex, insurance status, facility type, primary site (OC or OP), clinical tumor (cT), clinical nodal (cN) stage, pathologic nodal (pN) stage, neck dissection status, and treatment. Neck dissection was defined as patients that underwent regional lymph node surgery with at least 18 lymph nodes examined. Age was reported as a binary variable (< 60 and 60 + years) for chi-square analysis and as a continuous variable for regression analysis. Insurance status groups were private insurance, Medicaid, Medicare, other government insurance, and no insurance. Facility types included comprehensive community cancer programs, community cancer program, academic/research program, and integrated network cancer program; these categories are defined by the NCDB. Surgical margin status was defined as positive or negative. High-grade tumors were defined as poorly differentiated or undifferentiated, as previously described [13, 17]. Treatment modalities evaluated were no treatment, surgery only, radiotherapy only, chemotherapy only, surgery with adjuvant radiation, and surgery with adjuvant chemoradiotherapy. Patients with other or unclear treatment regimens were not excluded from descriptive analyses but were excluded in relevant regression analyses.

Patients undergoing neck dissection were compared with those not undergoing neck dissection with the chi-square test and independent samples *t* test for categorical and continuous variables, respectively. The primary outcome for this study was overall survival (OS). Kaplan-Meier and multivariable Cox regression proportional hazards model were implemented for univariate and multivariate survival analyses, respectively. Statistical significance was set at *p* < 0.05. All statistical analyses were performed utilizing SPSS version 26 (IBM Corp, Armonk, New York).

## Results

A total of 243 patients satisfied inclusion criteria, of which 79 (32.5%) underwent neck dissection (Table 1**)**. The majority of patients were less than 60 years old (60.1%), White (76.2%), male (53.5%), had private insurance (51.7%), oral cavity disease (60.9%), and underwent treatment at an academic center (60.6%). 146 (60.1%) patients had cN0 disease and 92 (37.9%) had cN + disease. Neck dissection was performed at academic institutions more frequently than non-academic institutions (73.2% vs. 53.8%, *p* = 0.014).

**Table 1:** Patient Clinical Characteristics

	No ND		ND		Total		*P*-value
	*N*	%	*N*	%	*N*	%	
**Total**	164	67.5	79	32.5	243		
**Age**							0.323
Mean, years (SD)	56.3 (17.6)		55.5 (13.3)		56.0 (16.3)		0.732
<60	95	57.9	51	64.6	146	60.1	
60+	69	42.1	28	35.4	97	39.9	
**Race**							**0.013**
White	120	74.1	62	80.5	182	76.2	
Black	40	24.7	10	13	50	20.9	
Other	2	1.2	5	6.5	7	2.9	
**Sex**							0.840
Male	87	53	43	54.4	130	53.5	
Female	77	47	36	45.6	113	46.5	
**Primary Site**							0.170
Oral Cavity	95	57.9	53	67.1	148	60.9	
Oropharynx	69	42.1	26	32.9	95	39.1	
**Insurance Type**							0.524
Private Insurance	82	51.9	40	51.3	122	51.7	
Not Insured	4	2.5	5	6.4	9	3.8	
Medicaid	19	12.0	10	12.8	29	12.3	
Medicare	51	32.3	23	29.5	74	31.4	
Other Government	2	1.3	0	0	2	0.8	
**Facility Type**							**0.014**
Community Cancer Program	2	1.5	3	4.2	5	2.5	
Comprehensive CCP	36	27.3	11	15.5	47	23.2	
Academic	71	53.8	52	73.2	123	60.6	
Integrated Cancer Program	23	17.4	5	7	28	13.8	
**N Stage**							**0.006**
N0	111	67.7	35	44.3	146	60.1	
N1	13	7.9	13	16.5	26	10.7	
N2	35	21.3	29	36.7	64	26.3	
N3	2	1.2	0	0	2	0.8	
Nx	3	1.8	2	2.5	5	2.1	
**Grade**							**0.010**
Well Differentiated	39	30.5	11	15.5	50	25.1	
Moderately Differentiated	59	46.1	28	39.4	87	43.7	
Poorly Differentiated	19	14.8	21	29.6	40	20.1	
Undifferentiated	11	8.6	11	15.5	22	11.1	
**Pathologic N Stage**							**< 0.001**
p0	29	20.4	29	37.2	58	26.4	
p1	5	3.5	4	5.1	9	4.1	
p2	10	7.0	32	41.0	42	19.1	
pX	98	69.0	13	16.7	111	50.5	
**Treatment Type**							**< 0.001**
No Treatment	18	14.0	1	1.3	19	9.3	
Surgery Only	48	37.2	22	29.3	70	34.3	
Radiation Only	17	13.2	0	0	17	8.3	
Surgery with aRT	33	25.6	40	53.3	73	35.8	
Surgery with aCRT	11	8.5	11	14.7	22	10.8	
Chemotherapy Only	2	1.6	1	1.3	3	1.5	
**Surgical Margins**							0.445
Negative Margins	96	79.2	62	83.8	138	81.2	
Positive Margins	20	20.8	12	16.2	32	18.8	

Abbreviations; SD: Standard Deviation; ND: Neck Dissection; aRT: Adjuvant Radiotherapy; aCRT: Adjuvant chemoradiotherapy; CCP: Community Cancer Program

Of 79 patients undergoing neck dissection, 35 (44.3%) had cN0 disease and 42 (53.2%) had cN + disease. Of 164 patients not undergoing neck dissection, 111 (67.7%) had cN0 disease and 53 (32.3%) had cN + disease. Patients with cN + disease underwent neck dissection more frequently than those with cN0 disease (53.2% vs. 30.5%, *p* = 0.002). Patients undergoing neck dissection more commonly had high-grade tumors than those not undergoing neck dissection (45.1% vs. 23.4%, *p* = 0.010). Upon examination of dissected lymph nodes, 13 (20.3%) cN0 patients undergoing neck dissection had occult nodal metastases (Table 2**)**.

**Table 2:** Patient Nodal Disease Characteristics

	Pathologic *N* Stage				
**Clinical N Stage**	p0		p+		Total
**All Patients**	N	%	N	%	N
cN0	50	79.4	13	20.6	63
cN+	7	15.9	37	84.1	44
cNx	1	50.0	1	50.0	2
**High-grade Patients**					
cN0	6	66.7	3	33.3	9
cN+	3	15.0	17	85.0	20

Of the 146 patients with cN0 disease, 55 (42.3%) underwent adjuvant therapy. Of 22 patients with cN0 disease and high-grade tumors, 14 (63.6%) underwent adjuvant treatment. 51 (68.0%) patients undergoing neck dissection also underwent adjuvant therapy; only 44 (34.1%) patients not undergoing neck dissection also underwent adjuvant therapy.

**Fig. 1:**
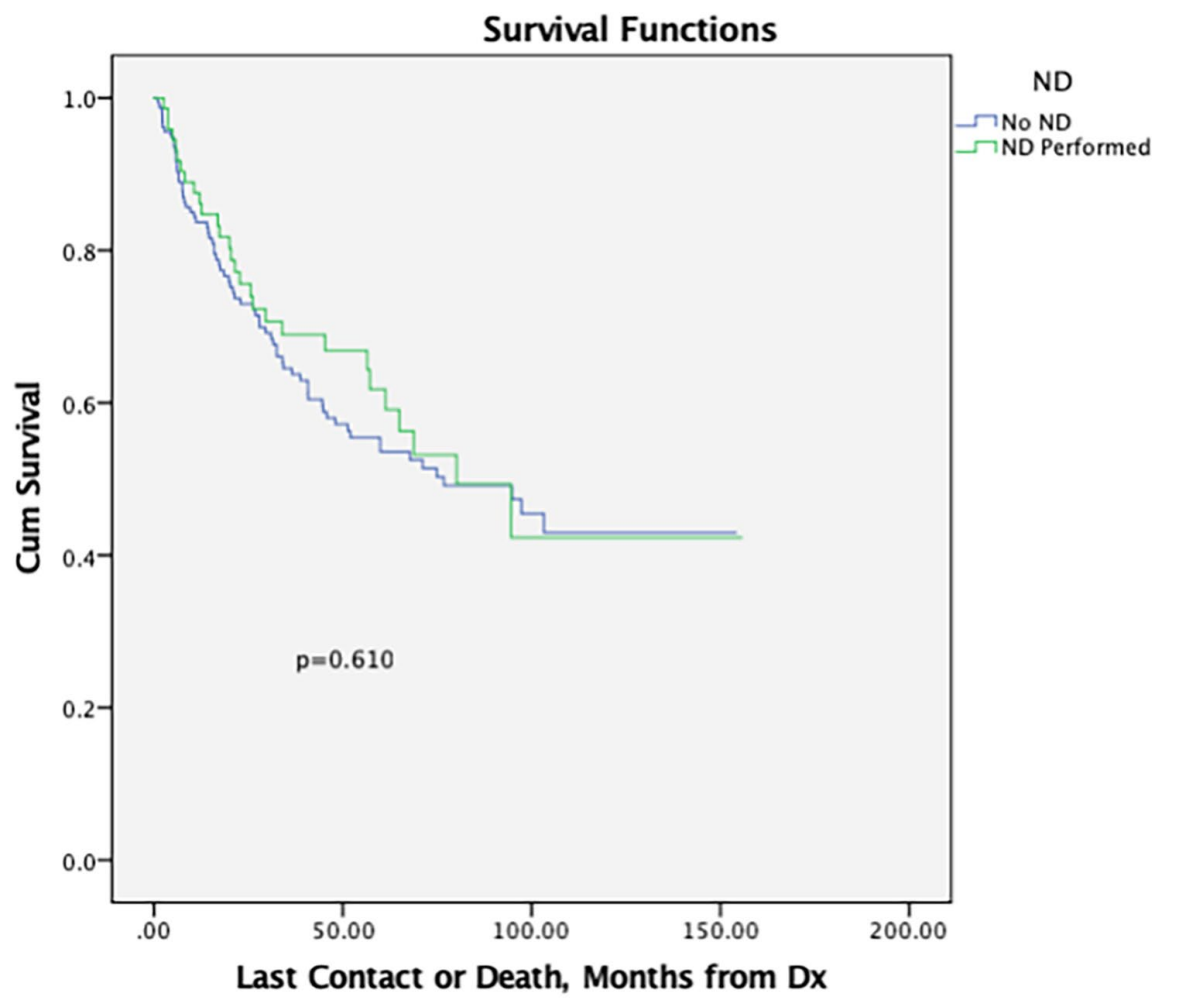
Kaplan-Meier survival curve for all patients treated with and without neck dissection

**Fig. 2:**
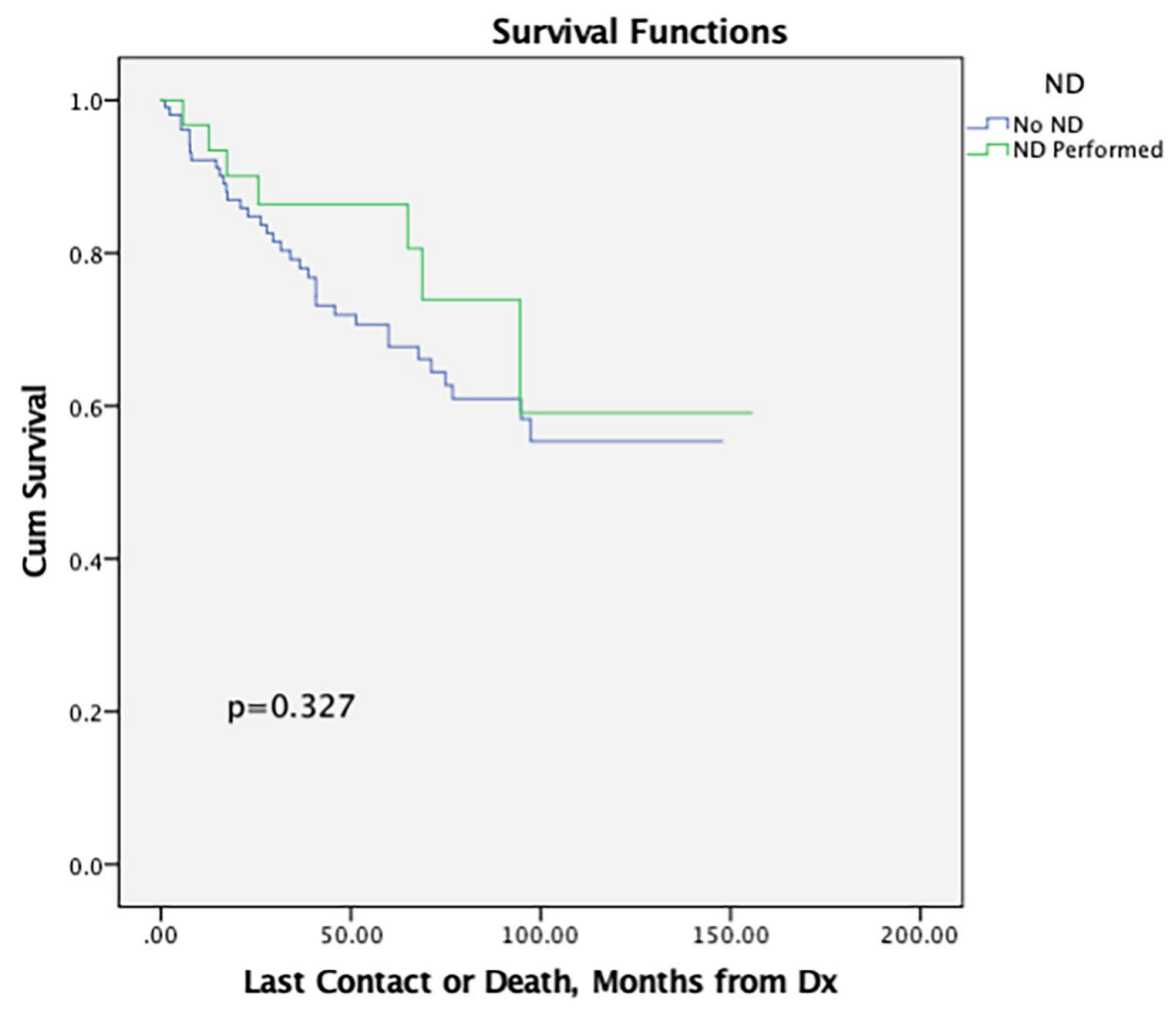
Kaplan-Meier survival curve for patients with cN0 disease treated with and without neck dissection

On Kaplan-Meier analysis, there was no significant difference in 5-year OS between patients undergoing and not undergoing neck dissection (61.8% vs. 53.6%, *p* = 0.610) (Table 3; Fig. 1**)**. Among patients with cN0 disease, those undergoing neck dissection similar 5-year OS as those not undergoing neck dissection (86.3% vs. 67.7%, *p* = 0.327) (Fig. 2**)**.

**Table 3:** Kaplan Meier Analysis for Impact of Neck Dissection on Survival

Variable	No. Patients	1-Year OS (%)	2-Year OS (%)	5-Year OS (%)	*p* value
**All Patients**					0.610
No ND	157	83.7	73.0	53.6	
ND Performed	73	87.6	75.6	61.8	
**cN0 Patients Only**					0.327
No ND	105	92.2	84.8	67.7	
ND Performed	31	96.8	90.1	86.3	
**cN + Patients Only**					0.091
No ND	49	66.8	47.8	22.7	
ND Performed	41	80.2	63.8	45.9	
**High-grade Tumors Only in All Patients**					0.724
No ND	30	63.3	48.4	36.3	
ND Performed	30	83.3	66.1	39.6	
**High-grade Tumors in cN0 Patients**					0.743
No ND	14	71.4	57.1	49.9	
ND	7	85.7	71.4	57.1	

Abbreviations: OS: Overall Survival; ND: Neck Dissection

Multivariable Cox regression proportional hazards model was implemented to evaluate the effect of neck dissection on survival in the context of other relevant clinicopathologic features (Table 4**)**. Increasing age (hazard ratio [HR]: 1.05, 95% confidence interval [CI]: 1.03–1.07, *p* < 0.001), poorly differentiated (HR: 4.10, 95% CI: 1.63–10.31, *p* = 0.003), and undifferentiated tumors (HR: 4.25, 95% CI: 1.29–13.99, *p* = 0.017) were significantly associated with 5-year OS. Neck dissection, however, was not significantly associated with 5-year OS (HR: 1.52, 95% CI: 0.73–3.18, *p* = 0.269).

**Table 4:** Cox Regression Proportional Hazards Model

Variable	HR	*p*-value	95% Confidence Interval	
			Lower	Higher
**Age**	1.05	**< 0.001**	1.03	1.07
**Race**				
White	REF			
Black	1.29	0.502	0.62	2.69
Other	0	0.979	0	.
**Sex**				
Male	REF			
Female	0.92	0.803	0.47	1.79
**Primary Site**				
Oral Cavity	REF			
Oropharynx	1.40	0.369	0.68	2.89
**Clinical N Stage**				
N0	REF			
N1	0.87	0.802	0.30	2.57
N2	2.80	**0.015**	1.22	6.43
N3	1.47	0.665	0.26	8.26
Nx	1.37	0.777	0.16	12.07
**Grade**				
Well Differentiated	REF			
Moderately Differentiated	2.11	0.099	0.87	5.11
Poorly Differentiated	4.10	**0.003**	1.63	10.31
Undifferentiated	4.25	**0.017**	1.29	13.99
**Neck Dissection**				
No ND	REF			
ND Performed	1.52	0.269	0.73	3.18
**Treatment Regimen**				
No Treatment	REF			
Surgery Only	0.22	**0.013**	0.07	0.73
Radiation Only	0.66	0.549	0.17	2.58
Surgery with aRT	0.11	**0.001**	0.03	0.39
Surgery with aCRT	0.37	0.139	0.10	1.38
Chemotherapy only	4.23	0.157	0.57	31.23

## Discussion

MEC of the OC and OP is a rare malignant tumor, contributing to the lack of randomized clinical trials and other high-quality evidence that can inform surgeons on the optimal management of these patients. Ellis et al. characterized prognostic factors and occult nodal metastasis for these patients using the 2004–2013 NCDB [13]. Their study elucidated the deleterious impact of increasing tumor size, nodal disease, and surgical margin status on survival. Similarly, they noted a 14.1% and 17.3% rate of occult disease in high-grade and cT3/4 tumors, respectively [13]. This study aimed to build upon their work and evaluate the benefit of neck dissection in these relatively high-risk patients. This analysis found that neck dissection was not significantly associated with improved survival but did report a higher rate of occult nodal metastasis (20.3%).

This study found that MEC of the OC and OP most occurred in White males in their mid-50s. These findings align with other studies which have corroborated an increased incidence of the OC and OP MEC in White individuals in their 50s [2, 6, 13, 18]. However, these analyses have reported a female predominance. Selecting for specifically cT3/T4 disease may be responsible as previous studies have found that males are more often diagnosed with higher grade and more advanced SGC [19, 20]. This may result in the majority male cohort. Surgical treatment with or without adjuvant therapy was the most common treatment modality (> 80%). The positive surgical margin rate for these patients was 18.8%. These findings are greater than those reported by Ellis et al., but lower than other studies analyzing MEC [13, 21, 22]. Ellis et al. reasoned that greater surgical access offered to tumors of the OC and OP compared to other locations in the upper aerodigestive tract likely translated to a lower positive margin rate [13]. Furthermore, this study only includes cT3/T4 tumors; the added bulk may contribute to this relatively higher positive margin rate as Ellis et. al’s study included cT1-4 MEC of the OC and OP.

This analysis found that 20.3% of patients with a clinically negative neck were found to have nodal disease on pathologic examination. The occult disease rate is higher than the 17.3% reported for cT3-4 MEC of the OC and OP by Ellis et al. and the 9.3% for MEC of the parotid gland reported by Xiao et al. [13, 23]. Cohort differences are likely a causal factor. For example, in an analysis of MEC of the parotid gland, Grasl et al. reported the risk for occult nodal metastases significantly increased with grade [24]. Other studies in the literature have characterized grade and other important risk factors such as tumor size and lymphovascular invasion as risk factors for occult nodal metastasis [13, 17, 24–26]. This cohort’s composition of only cT3/T4 patients and approximately 30% high-grade tumors (compared to 13.0% for Ellis et al.) likely drives the difference in occult nodal metastasis [13]. It is important to note that 33.3% of cN0 patients with a high-grade cT3/T4 tumor had occult nodal metastasis. Unfortunately, due to rarity of these cancers, the low sample size of this cohort does limit this finding’s utility (*n* = 9). Furthermore, in comparison to other studies analyzing MEC of alternate primary sites, it is important to consider the anatomic architecture of the OC and OP. Specifically, the OC and OP have rich lymphatic supplies that may result in higher risk of nodal metastases and more advanced disease burden [27, 28]. These findings indicate that elective neck dissection should be given strong consideration for patients with cT3/4 N0 MEC of the OC and OP.

The utility of elective neck dissection for SGC is controversial and necessitates consideration of many factors such as associated surgical morbidity, tumor histology, and adverse features [25]. On Kaplan-Meier analysis, while there did appear to be higher survival rates for patients undergoing neck dissection versus not, regardless of neck nodal status, there was no significant survival benefit for patients undergoing neck dissection. These findings align with several reports in the literature. Studying patients with cT1-2 N0 parotid MEC, Al-Qurayshi et al. found that elective neck dissection did not confer a survival benefit [25]. Nance et al. reported for MEC of the head and neck that the association of neck dissection with survival varied with tumor grade, finding no survival benefit for intermediate grade tumors and decreased survival in their high-grade group [29]. Herman et. al’s study of 59 patients with cN0 high-grade SGC did not find a difference in survival or locoregional control for patients treated with elective neck irradiation versus elective neck dissection. Similarly, analyzing cN0, high-grade parotid cancer, Harbison et al. also reported no statistically significant survival benefit for patients undergoing elective neck dissection, suggesting that radiation or elective neck dissection can be viable options to manage occult nodal metastases [30]. This study similarly failed to detect a significant difference in survival. A possible reason for this may be the use of adjuvant therapy in cN0 patients. Sample size limitations may also be a factor as the rarity of cT3/4 MEC of the OC and OP could have resulted in statistical underpowering, leading to a failure to detect a survival difference. As such, physicians should utilize patient-specific factors such as tumor size and grade when evaluating the decision for elective neck dissection as patients with larger and high-grade tumors appear to be at increased risk for occult nodal metastasis. Further prospective studies are necessary to better understand the optimal management of cN0 MiSGC.

Certain limitations inherent to all retrospective studies utilizing a national database need to be considered. Cases may have inaccurate or missing data, which are unverifiable due to lack of access to the original patient charts. Moreover, the NCDB is limited by the number of available variables; as such, there are likely potential confounders or important factors which our analysis could not capture such as specific comorbidities, tobacco use, imaging, quality of life, and multidisciplinary tumor board recommendations. In addition, certain studies have proposed the prognostic significance of several molecular markers in MEC which cannot be accounted for in this analysis [6]. We also are unable to analyze other relevant outcome variables such as disease-specific mortality, locoregional control, and recurrence-free survival. Despite these limitations, the NCDB has proven to be an invaluable resource for understanding survival outcomes of rare head and neck cancers.

## Conclusion

The majority of patients with cT3/4 MEC of the OC and OP presented with cN0 disease, with a high rate of occult nodal metastasis. Patients with cN + disease and high-grade tumors more frequently underwent neck dissection. Neck dissection was not significantly associated with 5-year OS, possibly because of sample size limitations. The statistical insignificance of neck dissection persisted among patients with cN0 disease, cN + disease, and high-grade tumors. Neck dissection, however, should be given strong consideration for high-grade and cT3/4 MEC of the OC and OP because of the high rate of occult nodal metastasis. Further research and prospective studies are needed to better inform the decision to pursue neck dissection in this patient population.
